# Antibody drugs conjugates in small-cell lung cancer: present-day status and promises

**DOI:** 10.1093/oncolo/oyaf332

**Published:** 2025-10-06

**Authors:** Arjun Syal, May-Lucie Meyer, Kenneth Angelino, Noah Osei, Jorge E Gomez, Fred R Hirsch, Triparna Sen

**Affiliations:** Department of Internal Medicine, Icahn School of Medicine, Mount Sinai Morningside and West, New York, NY 10019, United States; Center for Thoracic Oncology, Icahn School of Medicine at Mount Sinai, New York, NY 10029, United States; Tisch Cancer Institute, Icahn School of Medicine at Mount Sinai, New York, NY 10029, United States; Department of Oncology, Centre Hospitalier Universitaire Vaudois (CHUV), Lausanne 1011, Switzerland; Center for Thoracic Oncology, Icahn School of Medicine at Mount Sinai, New York, NY 10029, United States; Tisch Cancer Institute, Icahn School of Medicine at Mount Sinai, New York, NY 10029, United States; Center for Thoracic Oncology, Icahn School of Medicine at Mount Sinai, New York, NY 10029, United States; Tisch Cancer Institute, Icahn School of Medicine at Mount Sinai, New York, NY 10029, United States; Center for Thoracic Oncology, Icahn School of Medicine at Mount Sinai, New York, NY 10029, United States; Tisch Cancer Institute, Icahn School of Medicine at Mount Sinai, New York, NY 10029, United States; Center for Thoracic Oncology, Icahn School of Medicine at Mount Sinai, New York, NY 10029, United States; Department of Internal Medicine, The Ohio State University, Columbus, OH 43210, United States; The Ohio State University Comprehensive Cancer Center—Arthur G. James Cancer Hospital and Richard J. Solove Research Institute, The Ohio State University, Columbus, OH 43210, United States

**Keywords:** small-cell lung cancer, thoracic oncology, biomarkers, antibody-drug conjugates, targeted therapy

## Abstract

**Introduction:**

Small-cell lung cancer (SCLC) accounts for 15% of lung cancers and is characterized by an aggressive disease course and historically poor prognosis. Although tumors in most patients respond to initial chemotherapy, relapse is nearly universal and treatment options remain limited. Antibody-drug conjugates (ADCs) have emerged as a novel therapeutic class with the potential to address this unmet need.

**Methods:**

ClinicalTrials.gov, PubMed, and their associated references and press releases were queried for the search terms “antibody-drug ­conjugates” and “SCLC.” Only English-language sources were included.

**Results:**

Multiple ADCs targeting diverse antigens have been evaluated in relapsed or refractory SCLC. Topoisomerase I inhibitor payloads have generated the most consistent activity across delta-like protein 3, TROP2, B7-H3, and SEZ6 targets, while microtubule and pyrrolobenzodiazepine-based constructs have not demonstrated durable benefit. Despite encouraging response rates, progression-free survival has remained short, reflecting intrinsic resistance, antigen heterogeneity, and toxicity-related dose limitations.

**Conclusion:**

While ADCs have generated encouraging response rates in SCLC, durability has remained limited. Future development will require optimization of payloads, linkers, and trial design to determine whether ADCs can achieve a sustained role in this disease.

Implications for Practice Historically, therapeutic progress in small-cell lung cancer (SCLC) has been modest. Antibody-drug conjugates (ADCs) offer the potential to reshape the treatment landscape. This review highlights principles of ADC design, current clinical data in SCLC, recent technological advances, and emerging directions. By synthesizing these insights, we aim to provide clinicians with a framework to interpret ongoing trials and anticipate how ADCs may be integrated into future SCLC treatment strategies.

## Introduction

Small-cell lung cancer (SCLC) accounts for approximately 15% of all lung cancers and is characterized by aggressive biology, early metastatic spread, and poor prognosis, causing an estimated 200 000 deaths globally each year.^1^^,^^2^ While tumors in most individuals have a strong initial response to first-line platinum-based chemotherapy, with or without immunotherapy, relapse is nearly universal in extensive-stage SCLC (ES-SCLC), and subsequent treatment options remain limited.^3^ Topotecan, approved in 1996 for the second-line setting, yielded response rates of only 16.9%-21.9%.^4^ For more than 2 decades, it remained the only FDA-approved option, until lurbinectedin received accelerated approval in 2020 and Tarlatamab was granted accelerated approval in 2024.^5^^,^^6^

Over the past decade, growing insights into SCLC biology have spurred interest in antibody-drug conjugates (ADCs)—engineered agents that couple a tumor-targeting antibody to a cytotoxic payload.^7^ By delivering chemotherapy directly to the antigen-expressing tumor cells, ADCs aim to increase intratumoral potency while mitigating off-target toxicity. Advances in antibody selection, cytotoxic payload, and linker chemistry offer opportunities to tailor ADCs to SCLC. Yet the fundamental biology of this disease including its rapid evolution, lineage plasticity, and intratumoral heterogeneity makes it a uniquely hostile setting for ADCs compared with tumors where target expression is more stable.^8^^,^^9^

The momentum in the field is evident: in 2024, HS-20093, an investigational B7-H3-targeted ADC linked to topoisomerase inhibitor, received US-FDA breakthrough therapy designation for patients with relapsed or refractory ES-SCLC.^10^ Concurrently, the development of other novel ADCs in the setting of SCLC has demonstrated immense potential to alter the treatment model for lung cancers.

In this review, we summarize the principles of ADC design and optimization, contextualize ADC development within the history of SCLC therapeutics, critically appraise the current clinical trial landscape, and discuss emerging strategies to overcome the unique biologic and pharmacologic hurdles of ADC use in SCLC.

## Materials and methods

This non–systematic review examines ADCs investigated for the treatment of patients with SCLC. Literature was identified through PubMed and Google Scholar searches using the terms “antibody-drug conjugates” and “SCLC.” Additional sources included clinicaltrials.gov, press releases, and references from identified publications. Relevant unpublished findings presented at major scientific meetings (ASCO, ESMO, and WCLC) were compiled from abstract books and the authors’ professional networks. The search was limited to English-language publications, and all cited sources underwent thorough evaluation.

### Mechanism of ADCs

ADC activity involves a multi–step process. The antibody binds to a specific cell-surface target and is internalized, after which the cytotoxic payload is released to induce cell death, typically through direct nuclear or cytoplasmic action. Payload-containing cellular debris can also damage neighboring cells via a bystander effect.^11-13^

The linker connecting antibody to payload is central to this process, balancing stability in circulation with efficient intratumoral release; designs are broadly classified as cleavable (chemical- or enzyme-sensitive) or non–cleavable, each with trade-offs in durability and release efficiency.^9^^,^^14^^,^^15^ The payloads themselves span several classes—including microtubule inhibitors (auristatins, maytansinoids), DNA-damaging agents such as pyrrolobenzodiazepine (PBD) dimers, and topoisomerase I inhibitors—each with distinct mechanisms and toxicity profiles. In SCLC, where dose intensity is often limited by toxicity and narrow therapeutic windows, improvements in both linker stability and payload design are expected to be as pivotal as antigen choice, with future directions focusing on less toxic yet more effective warheads, including immune-modulatory payloads and strategies for tumor-restricted activation.^16^

### ADCs: Current status of ADCs in SCLC treatment

Several ADCs have been evaluated in SCLC over the past few years, with varying activity across clinical trials. Below, we summarize the most advanced efforts by target, with key efficacy data outlined in Table 1.

**Table 1.** Antibody-drug conjugates (ADCs) in small-cell lung cancer (SCLC).

ADC name	Target	Phase (Trial)	Payload	Population	ORR (%); PFS (months)	Grade ≥3 AEs (%)	Development status
**Rova-T (rovalpituzumab tesirine)**	DLL3	Phase II (Trinity)	PBD dimer	ES-SCLC, ≥3L (DLL3-expressing, >1% by IHC)^a^	12.4; 3.5	63	Discontinued (TAHOE Phase III terminated for inferior OS and toxicity)
**SC-002**	DLL3	Phase I	PBD dimer	SCLC (limited or extensive) or LCNEC, ≥2L^b^	14; NR	>60	No further development planned
**ZL-1310**	DLL3	Phase 1a/1b	Topo-I	ES-SCLC, ≥2L	67 (79 at 1.6 mg/kg)^c^; NR	6 (<2.0 mg/kg); 23 overall	Ongoing; FDA fast track; registrational trial planned
**Sacituzumab govitecan (SG)**	TROP2	Phase II	Topo-I (SN-38)	ES-SCLC, ≥3L (post–platinum + ICI)	41.9; 4.4	74.4^d^	Ongoing; FDA breakthrough therapy designation (2024)
**SHR-A1921**	TROP2	Phase I	Topo-I	ES-SCLC, ≥2L^e^	33.3; 3.8	35.3	Ongoing (phase I)
**Infinatamab deruxtecan (I-DXd)**	B7-H3	Phase II (IDEATE-lung-01)	Topo-I	ES-SCLC, ≥2L	26.1 (8 mg/kg), 54.8 (12 mg/kg); PFS 4.2, 5.5	43.5 (8 mg/kg), 50.0 (12 mg/kg)^d^	Ongoing (phase 3, IDEATE-Lung02)
**HS-20093**	B7-H3	Phase I (ARTEMIS-001)	Topo-I	ES-SCLC, ≥2 L post– (platinum ± ICI)^f^	61.3 (8 mg/kg), 50.0 (10 mg/kg); PFS 6.4, 8.9	39.3 (neutropenia)	Ongoing; FDA breakthrough therapy designation (2024); global phase 1/2 planned
**Lorvotuzumab mertansine (IMGN901)**	CD56	Phase I (with carboplatin/etoposide)	DM1 (microtubule inhibitor)	ES-SCLC, 1L^g^	No improvement vs. chemo	88.3 LM + chemo; 70.2 chemo-only	Discontinued (added toxicity, no clinical benefit)

*Abbreviations:* ADC, antibody-drug conjugates; AEs, adverse events; DLL3, delta-like protein 3; ES-SCLC, extensive-stage SCLC; LCNEC, large-cell neuroendocrine carcinoma; ORR, objective response rate; PBD, pyrrolobenzodiazepine; SCLC, small-cell lung cancer; TEAEs, treatment emergent adverse events.

a TRINITY required DLL3-expressing tumors (>1% by IHC).

b SC-002 phase 1b expansion required DLL3-high tumors (≥75% by IHC).

c uORR, unconfirmed objective response rate (ZL-1310).

d Reported as TEAEs, not TRAEs.

e SHR-A1921 enrollment independent of TROP2 expression.

f HS-20093 enrolled regardless of tumor B7-H3 expression.

g Lorvotuzumab mertansine was evaluated in CD56-positive tumors in combination with platinum/etoposide.

#### DLL3

Delta-like protein 3 (*DLL3*) is an atypical inhibitory ligand of the *NOTCH* receptor, normally expressed at low levels in healthy adult tissue but highly enriched in neuroendocrine SCLC.^17^ Its restricted expression pattern and association with tumorigenesis made it an attractive target for ADCs.^18^ However, DLL-3-directed ADC development has faced key limitations at the intersection of target biology and drug engineering.

The most advanced anti–*DLL3* ADC to date was rovalpituzumab tesirine (Rova-T), a humanized anti–*DLL3* monoclonal antibody linked to a PBD dimer toxin. In the phase 2 TRINITY trial of patients with SCLC after ≥2 prior lines, the objective response rate (ORR) was 12.4% (95% CI: 9.1-16.4) overall, 14.3% (95% CI: 10.1-19.4) in individuals with *DLL3*-high tumors, and 13.2% (95% CI: 9.5-17.7) in individuals with *DLL3*-positive tumors, with grade 3-5 adverse events (AEs) in 63% of patients.^4^ The phase 3 TAHOE trial, comparing Rova-T to topotecan in high expressing *DLL3* metastatic SCLC in the second-line setting, was stopped early for shorter overall survival with Rova-T (median OS 6.3 vs. 8.6 months; hazard ratio [HR]: 1.46, 95% CI: 1.17-1.82) and high toxicity (grade 3-4 AEs in 42%, grade 5 AEs in 22%).^19^ The phase 3 MERU maintenance trial after first-line therapy showed no survival benefit and was terminated early.^20^

SC-002, another PBD-based *DLL3* ADC, demonstrated partial tumor responses in 14% of patients with SCLC or large-cell neuroendocrine carcinoma in a phase 1 trial (*n* = 35) but was associated with serious adverse events in 66%, leading to discontinuation of development.^21^ More recently, attention has shifted toward alternative payload classes. ZL-1310 is an anti–*DLL3* ADC linked to a camptothecin-derived topoisomerase I inhibitor. In the ongoing phase 1a/1b trial (*n* = 74), the unconfirmed ORR was 67%, with the 1.6 mg/kg cohort showing the most favorable activity (79%). Tumor responses were also observed in individuals with brain metastases and in those previously treated with DLL3-directed therapy. Grade ≥3 treatment-related adverse events (TRAEs) occurred in 6% at <2.0 mg/kg vs. 23% overall, including rare ILD at higher doses. ZL-1310 has received FDA fast track designation, and a randomized registrational trial in patients with 2L ES-SCLC is planned.^22^

Taken together, these results show that even in *DLL3*-high disease, ADCs have produced only modest response rates and short progression-free survival, insufficient to ensure benefit. The discontinuation of Rova-T and ABBV-011 highlighted that failure was driven not by lack of *DLL3* targetability but by the narrow therapeutic index of PBD payloads, which constrained dosing. ZL-1310, a topoisomerase-inhibitor-based *DLL3* ADC, achieved higher ORRs in early studies, confirming that payload class influences initial efficacy. Yet progression remained rapid, underscoring that internalization and payload delivery, while necessary, are insufficient on their own; heterogeneity and therapy-induced *DLL3* loss ultimately preclude durable benefit. This contrasts with tarlatamab—a T-cell engaging agent—which bypasses payload delivery entirely.^23^^,^^24^ This discrepancy explains why objective responses may appear generous in early analyses, yet progression-free survival remains short. Payload classes with a wider safety margin and strong bystander effect, such as camptothecin-derived topoisomerase I inhibitors, may better accommodate SCLC’s heterogeneity, whereas anti–metabolites, which have consistently shown minimal activity in this disease, are unlikely to add value.^25-27^ In contrast, *DLL3* × CD3 bispecific T-cell engagers such as tarlatamab bypass payload delivery altogether, enabling serial T-cell–mediated killing that can sustain activity despite heterogeneous antigen expression.^28^

#### TROP2

Sacituzumab govitecan (SG) is a *TROP2*-directed ADC composed of a humanized monoclonal antibody linked via a hydrolyzable linker to SN-38, an active metabolite of irinotecan. In the phase II TROPiCS-03 trial, treatment with SG resulted in an ORR of 41.9% (95% CI: 27.0-57.9) in the ES-SCLC cohort (*n* = 43), with a progression-free survival (PFS) of 4.4 months (95% CI: 3.8-6.1).^29^ Overall, 74.4% of patients experienced grade ≥3 treatment-emergent adverse events (TEAEs). In subgroup analyses, an ORR of 35.0% (15.4-59.2) in platinum-resistant disease (chemotherapy free interval [CTFI] < 90 days; median PFS 3.8 months, 95% CI: 1.4-7.6) and in 47.8% (95% CI: 26.8-69.4) in platinum-sensitive disease (CTFI ≥ 90 days; median PFS 5.0 months, 95% CI: 4.1-7.4).^29^ In late 2024, the US-FDA granted breakthrough therapy designation to SG based on the results from the TROPiCS-03 study.^30^

SHR-A1921 is a novel ADC composed of a humanized anti–*TROP2* IgG1 monoclonal antibody linked to a DNA topoisomerase I inhibitor via a tetrapeptide-based cleavable linker.^31^ In a study involving patients with ES-SCLC who had received multiple prior lines of therapy (*n* = 17), treatment with SHR-A1921 produced an ORR of 33.3% (95% CI: 15.2-58.8) and median PFS of 3.8 months (95% CI: 1.4-NR), including in tumors with low *TROP2* expression. Grade ≥3 TRAEs occurred in 6 patients, with stomatitis being the most common.^31^

In sum, these results show that *TROP2* ADCs can produce meaningful tumor responses in relapsed SCLC, but PFS remains short in both platinum-sensitive and platinum-resistant subgroups. ORR was numerically higher in platinum-sensitive disease, though this difference was not statistically demonstrated; the similar PFS suggests rapid emergence of resistance irrespective of initial sensitivity. Both SG and SHR-A1921 utilize topoisomerase-I payloads with bystander effect, which may better align with the proliferative biology of SCLC than microtubule or anti–metabolite payloads. However, resistance may arise through upregulation of ATP-binding cassette transporters that reduce intracellular SN-38 accumulation or through mutations or reduced expression of topoisomerase I that impair payload binding and activity.^32^^,^^33^ These mechanisms mirror those seen with conventional irinotecan, underscoring the need to leverage SCLC’s historical drug biology when selecting payloads. The activity of SHR-A1921 in tumors with low *TROP-2* expression likely reflects the potency of its topoisomerase inhibitor payload and the bystander effect, which can extend killing to adjacent tumor cells with lower target expression, albeit with a risk of increased off-tumor toxicity. Combinations targeting DNA damage response, such as SG with the ATR inhibitor berzosertib, aim to address these limitations.^34^

#### B7-H3

*B7-H3* is an immune checkpoint molecule expressed in many malignancies, including SCLC, with overexpression linked to poor prognosis.^35^^,^^36^

Infinatamab deruxtecan (I-DXd) is a humanized anti–*B7-H3* IgG1 conjugated to the topoisomerase I inhibitor deruxtecan (Dxd).^37^ In the phase 2 IDEATE-Lung01 study, 88 patients with relapsed SCLC (≥1 prior line of platinum-based chemotherapy and ≤3 prior lines of systemic therapy, Eastern Cooperative Oncology Group performance status (ECOG PS) 0-1) received 8 mg/kg (*n* = 46) or 12 mg/kg (*n* = 42) every 3 weeks.^37^^,^^38^

ORR was 26.1% and 54.8%, with median PFS of 4.2 and 5.5 months, respectively. Despite higher activity at 12 mg/kg, PFS remained modest, consistent with rapid resistance development. Grade ≥3 TEAEs were reported in 43.5% and 50.0% of patients in the 8 and 12 mg/kg cohorts, respectively; gastrointestinal and hematologic events predominated, and most ILD events were grade 1-2. The 12 mg/kg dose was selected for phase 3 evaluation (IDeate-Lung02; NCT06203210).^38^

HS-20093, an anti–*B7-H3* ADC with a topoisomerase I inhibitor (exatecan-derivative) payload achieved ORRs of 61.3% at 8 mg/kg and 50.0% at 10 mg/kg in the phase I ARTEMIS-001 study in patients with ES-SCLC who had prior platinum-based therapy with or without immunotherapy. Median PFS was 6.4 and 8.9 months, and grade ≥3 neutropenia occurred in 39.3% of patients.^39^ No correlation was observed between *B7-H3* expression and objective response, though patients whose tumors had immunohistochemistry (IHC) expression ≥1% showed a trend toward longer PFS. The lack of correlation with IHC expression further suggests that payload potency and bystander effect, rather than biomarker threshold, may dominate efficacy in SCLC, a pattern seen across multiple ADC targets. Based on these results, the US-FDA granted Breakthrough Therapy Designation for relapsed or refractory ES-SCLC.

Miroztamab clezutoclax, an anti–*B7-H3* antibody with a BCL-X_L_ inhibitor payload, demonstrated no confirmed tumor response in 14 patients with SCLC; fatigue, AST elevation, diarrhea, and headache (*n* = 3, each).^40^

DS-7300, also using an exatecan-derived topoisomerase I inhibitor, demonstrated an ORR of 53% in early SCLC evaluation.^41^

Overall, *B7-H3* is a broadly expressed and prognostically adverse target in SCLC, and early signals suggest topoisomerase I payloads may be more active. However, short PFS despite high ORRs highlights the need to address resistance mechanisms and optimize payload selection. As with DLL3 and TROP2, responses have not translated into durable disease control, underscoring the broader challenge of achieving sustained benefit with ADCs in SCLC.

#### SEZ6

*SEZ6* is a transmembrane protein expressed in SCLC and other neuroendocrine tumors. ABBV-706, an anti–*SEZ6* ADC with a topoisomerase I inhibitor payload, has demonstrated high preclinical activity in SCLC, neuroendocrine neoplasms, and CNS tumors.^42^ In a phase 1 study of ABBV-706 as monotherapy or in combination with budigalimab, carboplatin, or cisplatin, 22 patients with SCLC were enrolled; confirmed ORR was 40% (6/15 evaluable patients).^42^ Efficacy by combination arm has not yet been reported. Across all tumor types, 57% experienced grade ≥3 TRAEs, primarily hematologic (anemia, neutropenia, and fatigue). Further evaluation of ABBV-706 is ongoing.^42^

ABBV-011, an anti–*SEZ6* ADC with a calicheamicin payload, was discontinued after phase 1 evaluation in SCLC, where the ORR was 19% in the overall cohort (*n* = 98) and 25% in the dose-expansion cohort (*n* = 40), with median PFS of 3.5 months in the latter and grade ≥3 TRAEs in approximately 65% of patients.^43^

*SEZ6* is a compelling neuroendocrine marker, and early ABBV-706 data suggest that topoisomerase I payloads may deliver higher initial activity than the calicheamicin-based ABBV-011, highlighting the impact of payload choice in SCLC. Still, the signal is preliminary, and durability remains limited, underscoring the recurring challenge of sustaining benefit with ADCs in this disease. Given the relatively high ORR with ABBV-706, it will be important to determine whether *SEZ6* expression thresholds, heterogeneity, or adaptive downregulation shape efficacy. More broadly, *SEZ6* illustrates both the opportunity of novel neuroendocrine antigens and the recurring challenge of overcoming resistance and toxicity to sustain benefit.

#### CD56

*CD56* (*NCAM1)* is overexpressed in 95% of SCLC cases and minimally in normal tissues, making it an appealing target.^44^^,^^45^ Lorvotuzumab mertansine, an anti–CD56 ADC with a DM1 microtubule inhibitor payload, was evaluated in untreated SCLC in combination with carboplatin/etoposide vs. carboplatin/etoposide alone. The combination achieved an ORR of 67.1% (95% CI: 55.8-77.1) compared with 59% (95% CI: 42.1-74.4) carboplatin/etoposide alone. Median PFS was similar in both study arms: 6.2 months (95% CI: 5.4-7.2) for the combination vs. 6.7 months (95% CI: 5.3-7.5) for chemotherapy alone (HR: 0.93; 95% CI: 0.58-1.51). Treatment-related toxicities, including peripheral neuropathy, led more patients to discontinue therapy, and the regimen was discontinued for lack of clinical benefit.^46^ DXC006, a preclinical anti–*CD56* ADC with a topoisomerase I inhibitor payload, has shown potent activity in SCLC models with a favorable safety profile, but no clinical data are available. *CD56* remains a biologically attractive marker; however, experience with lorvotuzumab highlights the importance of payload selection and tolerability, as microtubule inhibitors may compound the neuropathy risk intrinsic to *CD56* targeting. Whether a topoisomerase-based strategy can exploit *CD56*’s broad expression while avoiding these liabilities will determine its clinical value.

### Recent advances and innovations

Beyond antigen choice, refinements in linker chemistry, payload selection, and antibody format have been central to optimizing ADCs in SCLC.

#### Linker technology

Newer approaches aim to reduce premature release and systemic toxicity, long-standing barriers in SCLC. Cyclobutane-1,1-dicarboxamide (cBu) linkers cleaved by cathepsin B may enhance tumor specificity, while silyl ether–based acid-cleavable linkers extend plasma half-life compared with traditional hydrazones.^15^^,^^47^^,^^48^ Fe(II)-reactive trioxolane (TRX) scaffolds, exploit abnormal iron metabolism but remain hampered by toxicity.^15^^,^^49^ Moreover, evaluation has largely been confined to late-line, refractory populations; earlier integration may be required to reveal the true potential of these designs. Poor vascularization and hypoxia may also constrain payload penetration, adding microenvironmental limits beyond antigen and payload.

#### Optimization of cytotoxic payloads

Payload selection has been the key determinant of both efficacy and tolerability in SCLC ADCs. Microtubule inhibitor payloads, though effective in other solid tumors, have shown little impact in SCLC, consistent with the limited efficacy of conventional anti–microtubule agents in this disease. By contrast, topoisomerase I inhibitors have produced the most promising signals across multiple targets (*DLL3*, *TROP2*, *B7-H3*, and *SEZ6*).^25^^,^^31^^,^^37^^,^^42^ Exatecan-derived scaffolds and other proprietary topoisomerase-inhibitor payloads have produced encouraging responses, including with DLL3-directed constructs, but progression-free intervals remain short relative to the depth of tumor regression. This disconnect between high response rates and modest durability underscores the central challenge for ADCs in SCLC and highlights the need for improved linker/payload engineering. In parallel, biomarker selection will be essential to identify patients most likely to benefit.^50^ In SCLC, highly variable and dynamic antigen expression has limited the reliability of target presence alone as a predictor of response. Lessons from NSCLC, where HER2 mutations and c-MET overexpression have defined ADC benefit, highlight the importance of predictive biomarkers beyond target presence alone.^51^^,^^52^ Internalization efficiency may also influence therapeutic sensitivity, though experience with Rova-T demonstrates that it is necessary but not sufficient to ensure durable benefit.

PBD dimers, highly potent DNA crosslinkers, have been constrained by a narrow therapeutic index in SCLC. Early PBD-based ADCs such as ROVA-T and SC-002 were discontinued due to dose-limiting toxicity despite strong preclinical potency.^4^^,^^21^ Other emerging payloads under exploration include DNA-damaging agents (eg, uncialamycin) and immune-stimulatory payloads (eg, TLR7/8 or STING agonists), which may help broaden ADC activity in this immunologically “cold” tumor type, though SCLC-specific clinical data are not yet available.^53-57^

In sum, topoisomerase inhibitor payloads remain the most compelling class in SCLC, but the critical need is not higher initial response rates, but strategies that extend the durability of benefit while maintaining an acceptable therapeutic index.

#### Antibody optimization and targeting of tumor vasculature

Antibody design can also influence delivery. Large IgGs penetrate poorly compared with small payloads, prompting exploration of single-chain and single-domain antibodies.^14^ Nanobodies are attractive for their stability and tissue penetration, though their rapid serum clearance often necessitates PEGylation or Fc fusion to restore half-life.^58-60^ Targeting tumor vasculature has also been attempted to improve delivery, but vascular-disrupting agents such as flavonoids have shown only mixed results.^61-65^ Whether engineering or microenvironmental modulation can ultimately overcome SCLC’s entrenched barriers of heterogeneity and resistance remains uncertain.

### Future perspectives

#### Bispecific antibodies

Bispecific antibodies, which engage 2 epitopes on the same antigen or 2 distinct antigens, may limit antigen escape and improve tumor coverage in SCLC, where heterogeneous and dynamic expression has constrained single-target ADCs.^66^^,^^67^ Agents such as PT217, a *DLL3* × *CD47* bispecific antibody, which received FDA fast track designation for patients with ES-SCLC,^68^ and ivonescimab, a bispecific PD-1 × VEGF antibody with promising initial activity in SCLC,^69^ illustrate the growing interest in dual-targeting approaches that combine enhanced tumor coverage with immune engagement. The key question is whether such designs will yield more durable benefit than conventional ADCs.

#### Probody-drug conjugates

Probody-drug conjugates (PDCs) use antibodies masked until activated by tumor-associated factors such as proteases or acidic conditions, aiming to reduce off-target effects.^62^^,^^70^ This conditional activation could make antigens with normal tissue expression feasible for targeting.^70^ Early trials across solid tumors suggest potential, though development remains technically complex and untested in SCLC.^66^

#### Proteolysis-targeting chimeras

Degrader antibody conjugates (DACs) link antibodies to proteolysis-targeting chimeras (PROTACs) rather than cytotoxins, harnessing the ubiquitin–proteasome system to degrade proteins.^71^ While conceptually distinct, this platform remains at the preclinical stage, and its feasibility in SCLC is unproven.^66^

#### Dual payloads

Dual-payload ADCs, designed to address tumor subpopulations with differing drug sensitivities, are appealing for a disease as heterogenous as SCLC.^72^ While no constructs have yet been tested clinically in SCLC, experience in other cancers shows mixed results, sometimes outperforming single-payload ADCs, other times adding toxicity without added benefit.^73-75^

#### Novel targets

Expanding the pool of targetable antigens remains a major need for ADC development in SCLC.

One candidate is junctional adhesion molecule 3 (*JAM3*), which shows high expression in preclinical SCLC models. An anti–*JAM3* monoclonal antibody conjugated to a toxin selectively inhibited *JAM3*-expressing, but not *JAM3*-silenced, SCLC cells, supporting its potential therapeutic relevance.^76^ Collectively, these innovations, including broadening target space, refining payload strategies, and re–engineering antibody platforms, aim to overcome the therapeutic index and durability challenges that have so far constrained ADCs in SCLC.

## Conclusion

Despite extensive investigation, ADCs have yet to establish a therapeutic role in SCLC. Across targets, responses have been observed but rarely translated into durable benefit, reflecting payload toxicity ceilings, antigen heterogeneity, and rapid resistance. Overcoming these barriers will require innovations in payload and linker design, coupled with lessons from prior development. Equally important will be the search for predictive biomarkers to identify patients most likely to benefit; lessons from NSCLC, where HER2 mutations and c-MET overexpression have defined ADC activity, illustrate the potential for biomarker-driven selection in lung cancer and eventually also in SCLC. Looking ahead, exploration of platforms such as bispecifics, probody designs, and dual-payload constructs may help determine whether ADCs can secure a lasting role in SCLC therapy.

## Data Availability

No new data were generated or analyzed for this review article.
